# NR4A1 counteracts JNK activation incurred by ER stress or ROS in pancreatic β‐cells for protection

**DOI:** 10.1111/jcmm.16028

**Published:** 2020-10-30

**Authors:** Ze‐qing Pu, Dong Liu, Hanse Pablick Patherny Lobo Mouguegue, Cheng‐wen Jin, Esha Sadiq, Dan‐dan Qin, Tian‐fu Yu, Chen Zong, Ji‐cui Chen, Ru‐xing Zhao, Jing‐Yu Lin, Jie Cheng, Xiao Yu, Xia Li, Yu‐chao Zhang, Yuan‐tao Liu, Qing‐bo Guan, Xiang‐dong Wang

**Affiliations:** ^1^ Department of Cell Biology Shandong University School of Medicine Jinan China; ^2^ Blood Transfusion Department Qilu Hospital of Shandong University Jinan China; ^3^ Department of Endocrinology Qilu Hospital of Shandong University Jinan China; ^4^ Department of Physiology Shandong University School of Medicine Jinan China; ^5^ Department of Endocrinology Qingdao Municipal Hospital Qingdao China; ^6^ Department of Endocrinology Shandong Provincial Hospital Affiliated to Shandong University Jinan China; ^7^ Key Laboratory of Protein Sciences for Chronic Degenerative Diseases in Universities of Shandong (Shandong University) Jinan China

**Keywords:** cbl‐b, ER stress, NR4A1 (Nur77), pancreatic β‐cells, p‐JNK, ROS

## Abstract

Sustained hyperglycaemia and hyperlipidaemia incur endoplasmic reticulum stress (ER stress) and reactive oxygen species (ROS) overproduction in pancreatic β‐cells. ER stress or ROS causes c‐Jun N‐terminal kinase (JNK) activation, and the activated JNK triggers apoptosis in different cells. Nuclear receptor subfamily 4 group A member 1 (NR4A1) is an inducible multi‐stress response factor. The aim of this study was to explore the role of NR4A1 in counteracting JNK activation induced by ER stress or ROS and the related mechanism. qPCR, Western blotting, dual‐luciferase reporter and ChIP assays were applied to detect gene expression or regulation by NR4A1. Immunofluorescence was used to detect a specific protein expression in β‐cells. Our data showed that NR4A1 reduced the phosphorylated JNK (p‐JNK) in MIN6 cells encountering ER stress or ROS and reduced MKK4 protein in a proteasome‐dependent manner. We found that NR4A1 increased the expression of cbl‐b (an E3 ligase); knocking down cbl‐b expression increased MKK4 and p‐JNK levels under ER stress or ROS conditions. We elucidated that NR4A1 enhanced the transactivation of cbl‐b promoter by physical association. We further confirmed that cbl‐b expression in β‐cells was reduced in NR4A1‐knockout mice compared with WT mice. NR4A1 down‐regulates JNK activation by ER stress or ROS in β‐cells via enhancing cbl‐b expression.

## 1 INTRODUCTION

Although the aetiology of type 2 diabetes is complex, it is well accepted that insulin resistance leads to sustained hyperglycaemia or hyperlipidaemia, which consequently results in cellular ER stress and/or ROS overproduction in pancreatic β‐cells, whereas ER stress or ROS causes cell damage or even apoptosis.^1^, ^2^, ^3^, ^4^, ^5^, ^6^ Pancreatic β‐cell loss plays an important role in the pathogenesis of type 2 diabetes. To reveal the mechanisms by which pancreatic β‐cells counteracts the apoptotic effect from ER stress or ROS may provide clues for β‐cell protection.

NR4A1 is an inducible multi‐stress response factor.^7^, ^8^, ^9^ It belongs to the NR4A family, and its molecular structure includes a transcriptional activation domain, a DNA‐binding domain and a putative ligand‐binding domain.^10^ So far, no nature ligand has been found in this family. Therefore, NR4A1 belongs to orphan nuclear receptors.^11^ NR4A1 acts as a transcriptional factor to enhance the expression of some genes without ligand binding.^12^, ^13^ NR4A1 participates in various physiological activities, including cell proliferation, differentiation and apoptosis.^14^, ^15^, ^16^, ^17^, ^18^ ER stress or ROS‐inducing NR4A1 expression in pancreatic β‐cells has been documented.^4^, ^5^ Controversy still exists regarding the role of NR4A1 in apoptosis. Some studies showed that it had anti‐apoptotic effects,^15^, ^19^, ^20^, ^21^, ^22^, ^23^, ^24^ whereas others showed it was pro‐apoptotic.^16^, ^18^, ^25^, ^26^, ^27^ A possible explanation for the above controversial conclusions may be accounted by the cellular location of NR4A1. When NR4A1 remains in the nucleus as a transcriptional factor, it enhances the expression of some anti‐apoptotic factors, such as WT1 and survivin ^4^, ^5^, ^24^; if NR4A1 is translocated into the cytoplasm, it associates with bcl2 and results in cell apoptosis.^27^

JNKs, also known as stress‐activated protein kinases, form an important subgroup of the mitogen‐activated protein kinase (MAPK) superfamily and have three isoforms (JNK1, JNK2 and JNK3). Upon activation by upstream kinases, the activated JNK has diverse cellular functions, including cell proliferation and differentiation under physiological conditions. The role of activated JNK on pro‐apoptosis has been described as in cell type–dependent manner.^28^ Cellular stresses, such as ER stress and oxidative stress (ROS), incur high level or sustained JNK activation, which further triggers apoptotic pathways in different cells.^29^, ^30^, ^31^, ^32^, ^33^, ^34^, ^35^, ^36^, ^37^, ^38^, ^39^ Our preliminary data showed that either TG (thapsigargin, an ER stress inducer) or H_2_O_2_ (hydrogen peroxide, a kind of ROS) increased the accumulation of phosphorylated JNK in pancreatic β‐cells, whereas overexpressing NR4A1 was able to reduce JNK phosphorylation. NR4A1 is a transcription factor. How can a transcript factor regulate JNK phosphorylation? To answer this question, we used pancreatic mouse insulinoma 6 cells (MIN6 cells) and NR4A1 KO mice to explore the underlying mechanism.

## 2 MATERIALS AND METHODS

### 2.1 Cell culture and reagents

MIN6 cells were obtained from ATCC and were cultured under the same conditions as described in the reference.^4^ NR4A1 overexpression cell lines (designated as OV cells) and control cell lines (designated as NC cells) were generated and maintained as described previously.^4^ cbl‐b knockdown cells (designated as KD‐cbl‐b cells) or the control cell clone (designated as CON‐cbl‐b cells) were cultured in the same medium as NC/OV cells plus G418 at 1 mg/mL.

DMEM was purchased from M&C GENE technology. Foetal bovine serum (FBS) was purchased from Gibco. TG was purchased from Sigma‐Aldrich. H_2_O_2_ was purchased from Sinopharm Chemical Reagent. Puromycin and G418 (sulphate) were purchased from InvivoGen. MG132 was purchased from MedChemExpress.

### 2.2 Mouse islet purification

NR4A1 knockout (KO) mice generated from C57BL/6J mice and wild‐type (WT) mice were purchased from Cyagen Biosciences. The genotypes of the new‐born mice were verified with PCR according to the manual from Cyagen Biosciences. Mouse islets were isolated with collagenase P (purchased from Roche Applied Science) as described previously.^40^ After separation, the islets were deposited by Hank's Balanced Salt Solution. Finally, the islets were selected under stereoscopic microscope. Animal experiments were carried out according to the Principles of Laboratory Animal Care by NIH. All experiment procedures were approved by the Animal Care and Use Committee of Shandong University School of Basic Medical Sciences. The Animal Care and Use Committee of Shandong University School of Basic Medical Sciences approved that all the experiment procedures fully complied with the Helsinki Declaration.

### 2.3 Lentiviral infection and stable cell line selection

Lentivirus encoding full‐length NR4A1 and control lentivirus were constructed and generated by GenePharma. MIN6 cells were infected with recombinant lentivirus encoding NR4A1 or control lentiviral vector, and stable cells were selected under puromycin selection.

Lentivirus encoding shRNA targeting to cbl‐b and control lentivirus were constructed in Obio Technology, and the targeting sequence was 5′‐AACACAGACGCCATGATTTGC‐3′.^41^ OV cells were infected with cbl‐b shRNA lentivirus or control scramble shRNA lentivirus, and the stable cells were selected under G418 drug pressure.

### 2.4 Plasmid construction

Mice genomic DNA was extracted as previously described.^42^ Mice cDNA library was obtained from WT C57BL/6J mouse liver with ReverTra Ace qPCR RT Kit (Toyobo). We designed a pair of primers with HindIII and XbaI restriction sites at 5′ and 3′ to amplify the cDNA of MKK4 and cloned the PCR product into pFLAG‐CMV™‐2 vector (Sigma). The sequence information of the primer is listed below:

Primer F: 5′‐CCCAAGCTTATGGTCCACAAACCAAGTGGG‐3′

Primer R: 5′‐GGGTCTAGATCAGTCGACATACATGGGCGAGC‐3′.

We designed four pairs of primers with Kpnl and XhoI restriction sites at 5′ and 3′, respectively, to amplify four different lengths of the cbl‐b promoter (1987, 1440, 981 and 485 bp) from mice genomic DNA. And then, the PCR products were cloned into pGL3‐basic luciferase reporter vector (Promega).

The pairs of primers used for the four different lengths of cbl‐b promoter are listed below:

Primer F (‐2008 bp): 5′‐CCC GGTACC GTCAGCCTACATAAACACTATC‐3′

Primer R (‐21 bp): 5′‐CCC CTCGAG TCGAACCAATCCTGAGA‐3′.

The primers used are 1440, 981 and 458 bp. They all shared the same reverse primer:

Primer F (‐1454 bp): 5′‐CCC GGTACC CCAGACTTGTACTTTACTGC‐3′

Primer F (‐995 bp): 5′‐CCC GGTACC TCACTGTAAGCGTTACCA‐3′

Primer F (‐499 bp): 5′‐CCC GGTACC TATTTGGCTGCTCTCCT‐3′

Primer R (‐14 bp): 5′‐CCC CTCGAG TTGAGTCTTCGAACCAA‐3′.

All constructed plasmids were sequenced by Sangon Biotech Co., Ltd., to verify these sequences were completely aligned with the original DNA sequences from NCBI.

### 2.5 Quantitative real‐time PCR assay

Total RNA was extracted and purified from cultured cells by using RNAiso Plus (Takara) and from mouse islets using a RNeasy Mini Kit (Qiagen). qPCR was performed as previously described.^4^ According to the Cq value of the target gene obtained from the qPCR machine, the relative expression multiple of the target gene was calculated with a relative quantitative method for 2^‐ΔΔCq^, and the numerical value was further corrected by using the housekeeping gene 18s rRNA as the internal control. All experiments were repeated three times, and each sample was assayed in triplicate. The primers used for PCR are listed in Table 1.

**Table 1:** Primers for real‐time quantitative PCR

Genes	Sequence (5′→3′)
18S rRNA F	CGCGGTTCTATTTTGTTGGT
18S rRNA R	AGTCGGCATCGTTTATGGTC
NR4A1 F	ATGCCTCCCCTACCAATCTTC
NR4A1 R	CACCAGTTCCTGGAACTTGGA
MKK4 F	AGGACTTGAAAGACCTTGGAGA
MKK4 R	TATCTGCCCACTTGGTTTGTG
MKK7 F	ATGGAGAGCATCGAGATTGACC
MKK7 R	TTGATTTCTGCCTGATAACGCT
Cbl‐b F	GGTCGCATTTTGGGGATTATTGA
Cbl‐b R	TTTGGCACAGTCTTACCACTTT
TRAF2 F	AGAGAGTAGTTCGGCCTTTCC
TRAF2 R	GTGCATCCATCATTGGGACAG
Smurf1 F	AGCATCAAGATCCGTCTGACA
Smurf1 R	CCAGAGCCGTCCACAACAAT
JNK F	GTGGAATCAAGCACCTTCACT
JNK R	TCCTCGCCAGTCCAAAATCAA

### 2.6 Western blot analysis

Protein samples were obtained, and Western blot analysis was performed according to the methods as previously described.^43^ The antibodies used in the experiment are as follows: rabbit antibodies, such as anti‐p‐JNK (1:1000), anti‐JNK (1:1000), anti‐MKK4 (1:1000) and anti‐cbl‐b (1:1000), were purchased from ProteinTech Group, Inc; anti‐GAPDH antibody (1:30 000) was purchased from Zsgb Bio; anti‐NR4A1 antibody (1:1000) were purchased from Affinity Biosciences and Abcam; and mouse anti‐beta actin (1:5000) and rabbit anti‐α‐Tubulin (1:5000) were purchased from Bioworld Technology.

### 2.7 Immunoprecipitation (IP)

Equal numbers of OV cells or NC cells were transfected with equal amount of pFLAG‐CMV™‐2‐MKK4 and pUb‐HA (a plasmid for overexpressing Ub with HA‐tag from Dr Chengjiang Gao's Lab, Shandong University School of Basic Medical Sciences). After 48 hourrs post‐transfection, the cells were harvested for immunoprecipitation as routinely used. The antibody used to pull down protein was anti‐flag monoclonal antibody from ProteinTech Group. The beads with protein A and protein G were purchased from Solarbio Science & Technology. The pull‐down samples were applied for Western blotting with 12CA5 (an anti‐HA monoclonal antibody).

### 2.8 Dual‐luciferase reporter assays

Plasmids with different lengths of cbl‐b promoter were co‐transfected into cells with TK (thymidine kinase promoter‐Renilla luciferase reporter) plasmid, or pGL3‐basic vector was co‐transfected into cells with TK plasmid as control. The luciferase activity was measured by a dual‐luciferase reporter assay kit (Vazyme Biotech Co., Ltd) after 48 hours post‐transfection.

### 2.9 Chromatin immunoprecipitation (ChIP) analysis

MIN6 cells were infected with adenovirus encoding NR4A1‐HA or control adenovirus. After 48 hours post‐infection, the infected cells were applied for ChIP assay. ChIP analysis was accomplished with a ChIP Assay Kit (Beyotime) as previously described.^4^ The specific anti‐HA monoclonal antibody (12CA5) was applied to pull down the DNA fragments associated with HA‐tagged NR4A1.

A pair of primers, 5′‐GCTGCTACTTTTTCAGTTCCTTTCCTCGTTC‐3′(F), and 5′‐TCCACGGTACACAATGGCCC‐3′ (R), were designed to amplify the specific target sequence (−251 to −118 bp) of the cbl‐b promoter (as indicated in Figure 6).

### 2.10 Immunofluorescence

The pancreatic tissues from WT mice or NR4A1 KO mice were fixed with paraformaldehyde (4%). The primary anti‐insulin mice antibody (Santa Cruz Biotechnology, Inc) and the primary anti‐cbl‐b rabbit antibody (ProteinTech Group, Inc) were applied for immuno‐staining. The overlapped colour (yellow) of insulin (red) with cbl‐b (green) in islets of pancreatic tissue indicated cbl‐b expression in pancreatic β‐cells.

### 2.11 Statistical analyses

Data were expressed as the mean ± SD. Statistical analysis was done with t‐test or analysis of variance (ANOVA) by using GraphPad Prism 5.0. A value of *P* < .05 (*) was considered a statistically significant difference.

## 3 RESULTS

### 3.1 NR4A1 down‐regulates JNK phosphorylation induced by TG or H_2_O_2_ in MIN6 cells

**Figure 1:**
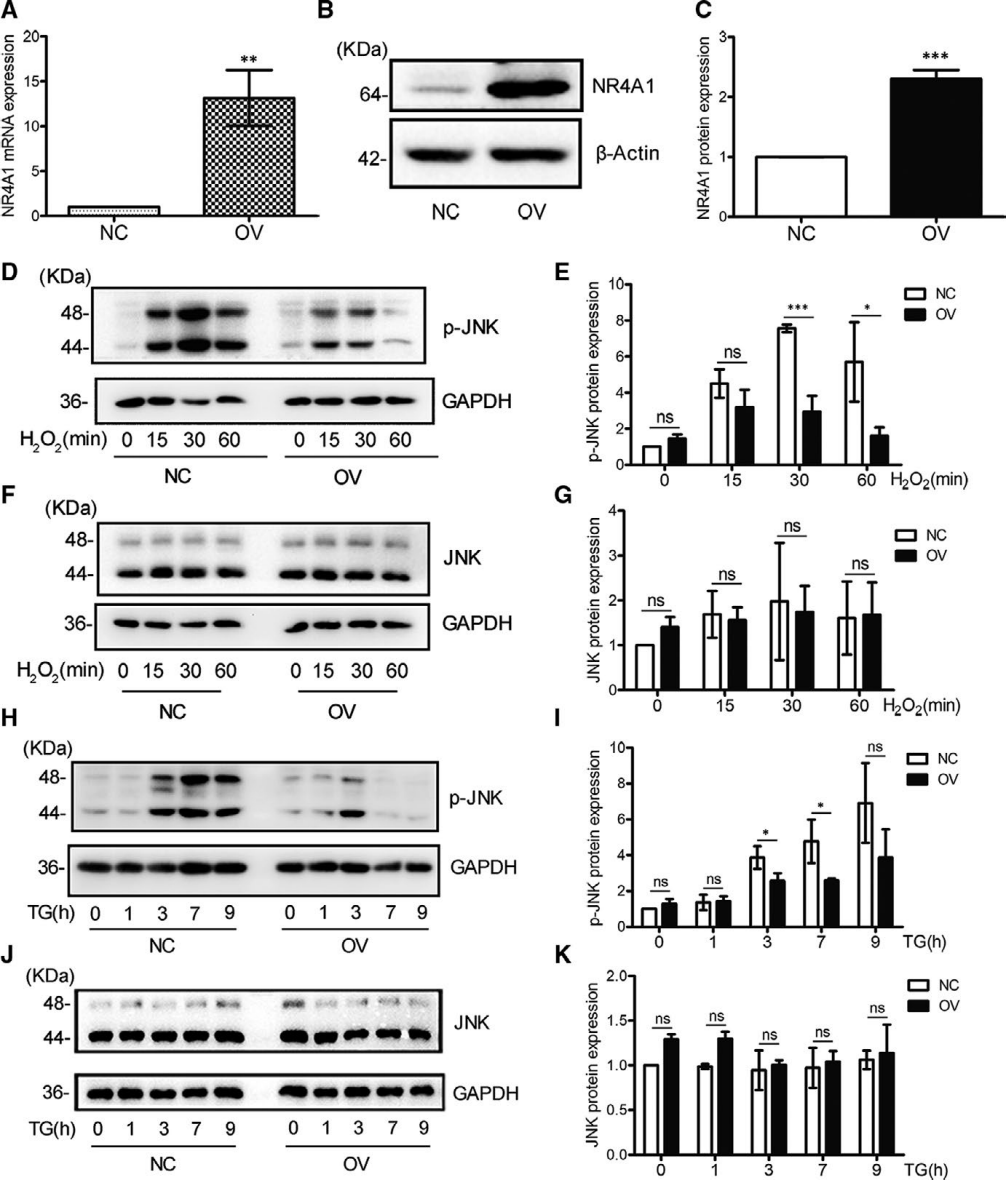
NR4A1 down‐regulates JNK phosphorylation induced by TG or H_2_O_2_ in MIN6 cells. A‐C, the relative NR4A1 mRNA and protein levels were determined by qPCR and Western blot in both OV (NR4A1 overexpression) and NC (control) cells. D‐G, Western blotting exhibited the protein levels of p‐JNK and JNK in response to 100 μmol/L H_2_O_2_ at various time‐points in both OV and NC cells. H‐K, Western blotting exhibited the protein levels of p‐JNK and JNK in response to 0.5 μmol/L TG at various time‐points in both OV and NC cells. Densitometric analyses of the blots were presented as histogram, and these data represented the means of three independent experiments; *P < .05, **P < .01 and ***P < .001 vs ns; error bars indicate SD

Previously, we generated NR4A1 overexpression cells (OV cells) from MIN6 cells and the control cells (NC cells). The mRNA and protein expression levels of NR4A1 were confirmed in OV cells and NC cells (Figure 1A‐C). Figure 1 showed that the phosphorylation level of JNK was increased in both OV cells and NC cells after they were treated with TG or H_2_O_2_, but overall the JNK phosphorylation level was lower in OV cells compared with that in NC cells (Figure 1D,E,H,I), whereas the total JNK level was close between the two cells (Figure 1F,G,J,K). Overexpressing NR4A1 turned down the JNK phosphorylation in MIN6 cells rather than total JNK level.

### 3.2 NR4A1 down‐regulates MKK4 protein level in MIN6 cells

**Figure 2:**
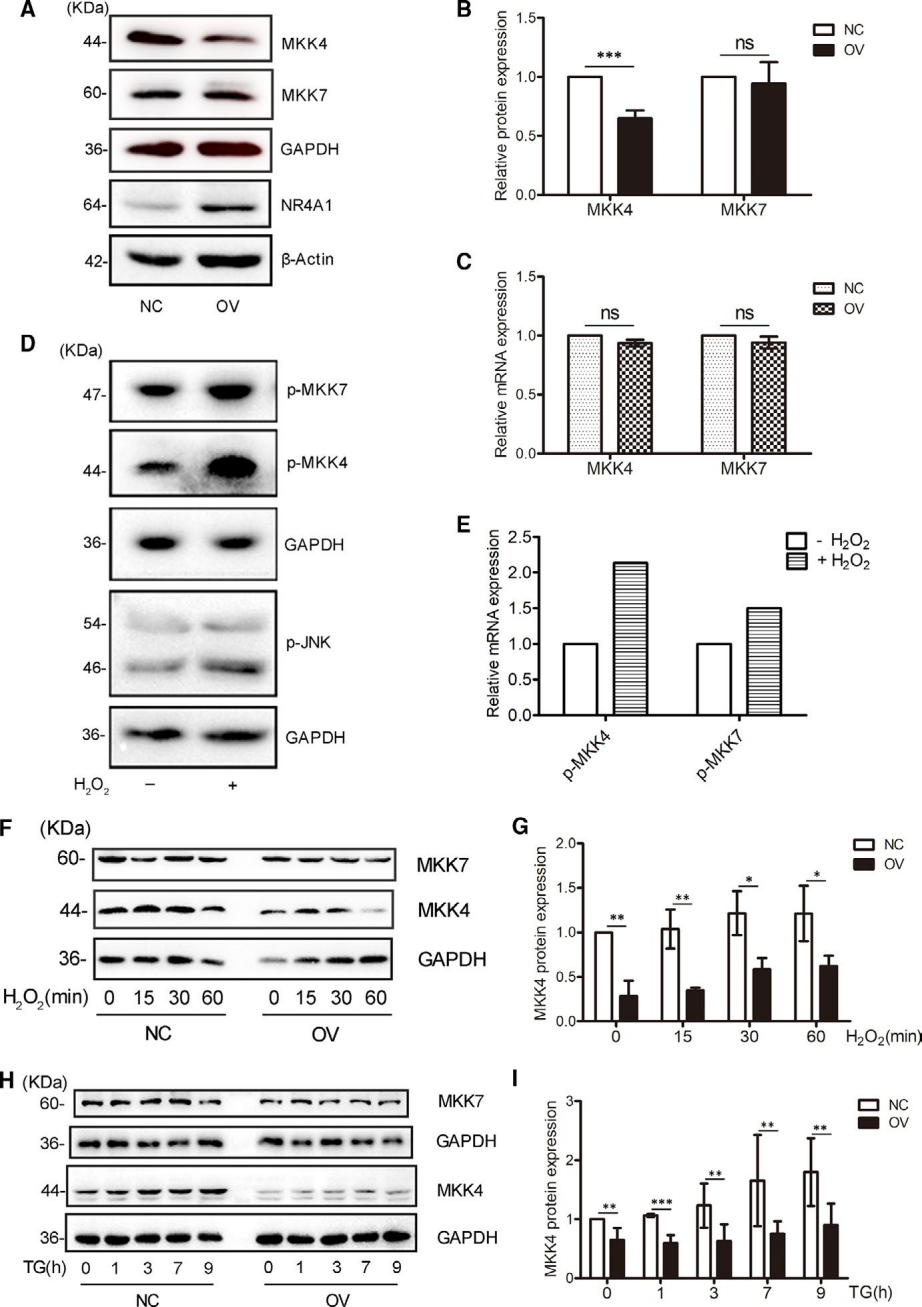
NR4A1 down‐regulates MKK4 protein level in MIN6 cells. A‐C, the relative mRNA and protein levels of MKK4 and MKK7 were determined by qPCR and Western blot in both OV and NC cells. D and E, MKK4 and MKK7 were verified to be phosphorylated during the treatment with H_2_O_2._ F and G, the protein levels of MKK4 and MKK7 in response to 100 μmol/L H_2_O_2_ at various time‐points in both OV and NC cells were determined by Western blot. H and I, the protein levels of MKK4 and MKK7 in response to 0.5 μmol/L TG at various time‐points in both OV and NC cells were determined by Western blot. Densitometric analyses of the blots were presented as histogram, these data represented the means of three independent experiments; *P < .05, **P < .01 and ***P < .001 vs ns; error bars indicate SD

To explore the mechanism by which NR4A1 attenuates JNK phosphorylation induced by ER stress or ROS, we searched online and found that there were two upstream kinase molecules specific for JNK, namely MKK4 and MKK7. The mRNA or protein level of MKK7 did not change between OV and NC cells (Figure 2A‐C), but the protein level of MKK4 in OV cells was markedly reduced compared with NC cells (Figure 2A,B), whereas the mRNA level of MKK4 did not change (Figure 2C).

To verify MKK4 and MKK7 are two upstream kinase molecules for JNK activation, the MIN6 cells were treated with H_2_O_2_ for 30 minutes, the phosphorylated MKK4(p‐MKK4) and phosphorylated MKK7(p‐MMK7) were detected with the specific antibodies, and the data showed that H_2_O_2_ increased the levels of both MKK4 and MKK7 phosphorylation levels. By comparison, the induced fold of p‐MKK4 was higher than that of p‐MMK7 (Figure 2D‐E).

The OV cells and NC cells were treated with TG or H_2_O_2_ at different time‐points, both MKK4 and MKK7 were examined in these samples, and we found that the MKK4 protein was maintained at a lower level in OV cells compared with that in NC cells during these series of treatments (Figure 2F‐I).

### 3.3 NR4A1 increases MKK4 ubiquitination and degradation in MIN6 cells

**Figure 3:**
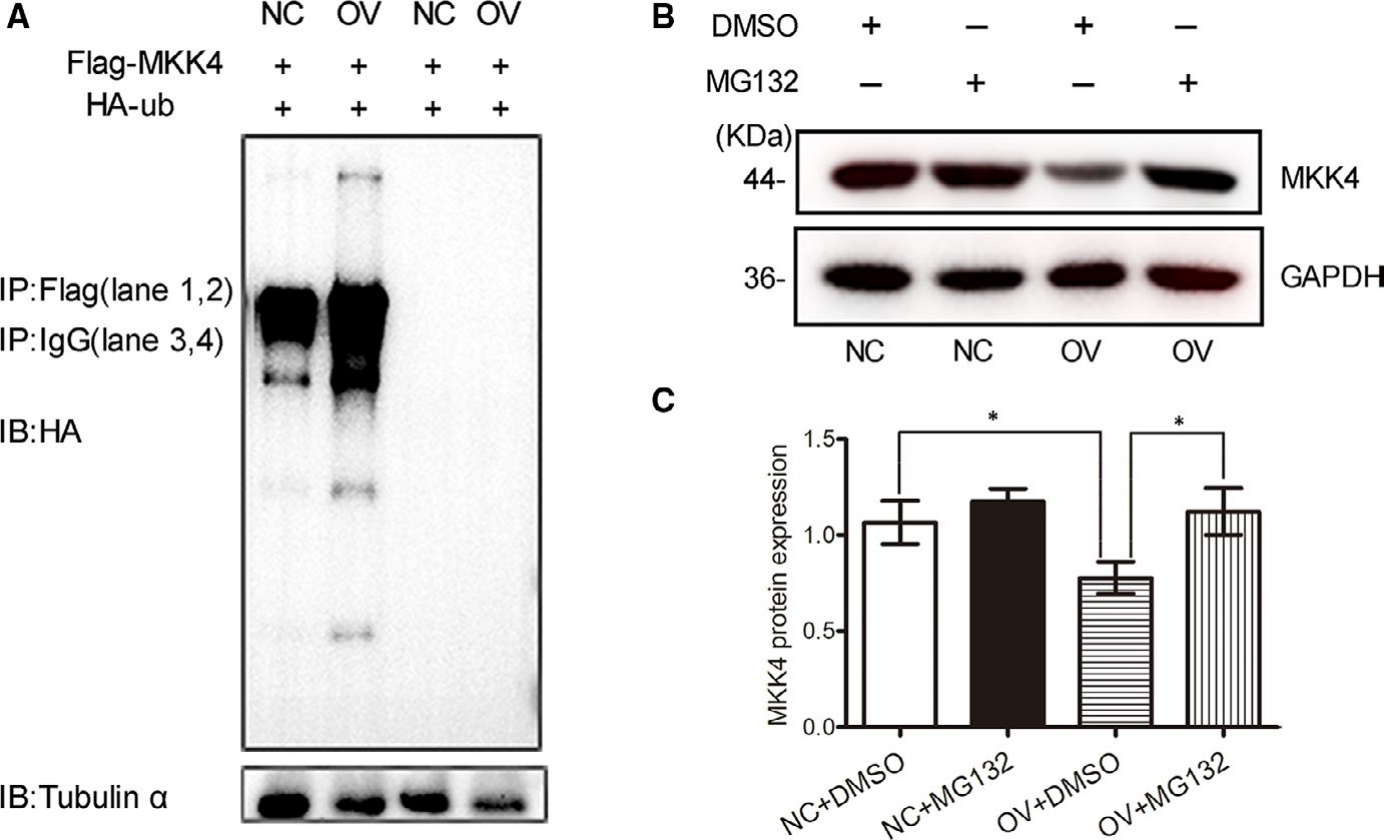
NR4A1 increases MKK4 ubiquitination and degradation in MIN6 cells. A, both OV cells and NC cell were transfected with two plasmids to express Flag‐MKK4 and HA‐ub. The Western blotting results showed the different ubiquitination levels of Flag‐MKK4 in OV and NC cells. B and C, OV cells or NC cells were treated with either 10 μmol/L MG132 or DMSO for 6 h. The protein level of MKK4 was detected with Western blotting in four different conditions. Densitometric analyses of the blots were shown as histogram; the data represented the means of three independent experiments; *P < .05 vs ns; error bars indicate SD

We tested whether overexpressing NR4A1 was able to enhance MKK4 ubiquitination level. In Figure 3, equal numbers of OV cells and NC cells were transfected with equal amount of Flag‐MKK4 and HA‐ubiquitin plasmids. The Flag‐MKK4 protein was pulled down with anti‐Flag monoclonal antibody. The results in Figure 3A showed that more ubiquitin modified exogenous Flag‐MKK4 in OV cells compared with that in NC cells. From the data above, it was reasonable to conclude that more overexpressed exogenous Flag‐MKK4 in OV cells was ubiquitinated compared with that in NC cells.

To further confirm that MKK4 was reduced by ubiquitination‐based proteasome degradation, MG132 was applied to figure out the dynamics of MKK4 in OV cells. As expected, MG132 was able to reverse the effect of NR4A1 down‐regulating MKK4 protein level in MIN6 cells (Figure 3B,C).

### 3.4 NR4A1 enhances the expression of cbl‐b in MIN6 cells

**Figure 4:**
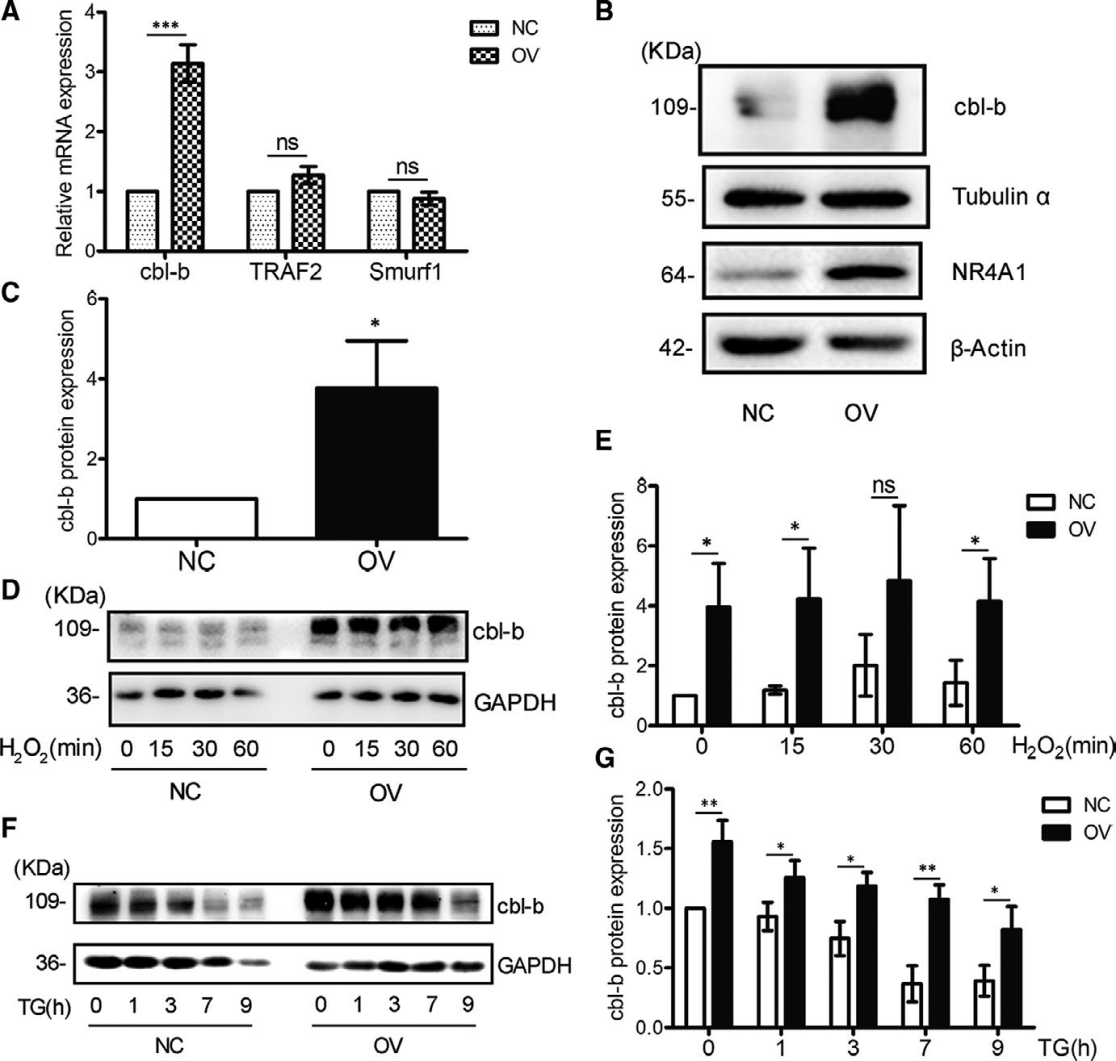
NR4A1 enhances the expression of cbl‐b in MIN‐6 cells. A, the relative cbl‐b mRNA levels were determined by real‐time quantitative PCR in both OV and NC cells. B and C, the relative cbl‐b protein levels were determined by Western blotting in both OV and NC cells. D and E, the protein levels of cbl‐b in response to 100 μmol/L H_2_O_2_ at various time‐points in both OV and NC cells were assessed by Western blotting. F and G, the protein levels of cbl‐b in response to 0.5 μmol/L TG at various time‐points in both OV and NC cells were assessed by Western blotting. Densitometric analyses of the blots are shown as histogram; the data represented the means of three independent experiments; *P < .05, **P < .01 and ***P < .001 vs ns; error bars indicate SD

Proteasome‐based protein degradation depends on the abundance of the specific E3 ligase. Each E3 ligase has a specific target or substrate to be modified with ubiquitin. It was reported that NR4A1 was able to enhance the expression of three possible E3 ligases, which were TRAF2,^19^ Smurf1 ^44^ and cbl‐b (casitas B‐lineage lymphoma b).^41^ Our data showed that OV cells had higher mRNA and protein levels of cbl‐b (Figure 4A‐C), whereas the mRNA levels of TRAF2 or Smurf1 did not increase in OV cells compared with NC cells (Figure 4A). During TG or H_2_O_2_ treatment for different time‐points, OV cells always maintained a higher level of cbl‐b protein expression compared with NC cells (Figure 4D‐G).

### 3.5 Cbl‐b expression negatively correlates with MKK4 protein level and p‐JNK level upon H_2_O_2_ or TG treatment in NR4A1‐overexpression cells

**Figure 5:**
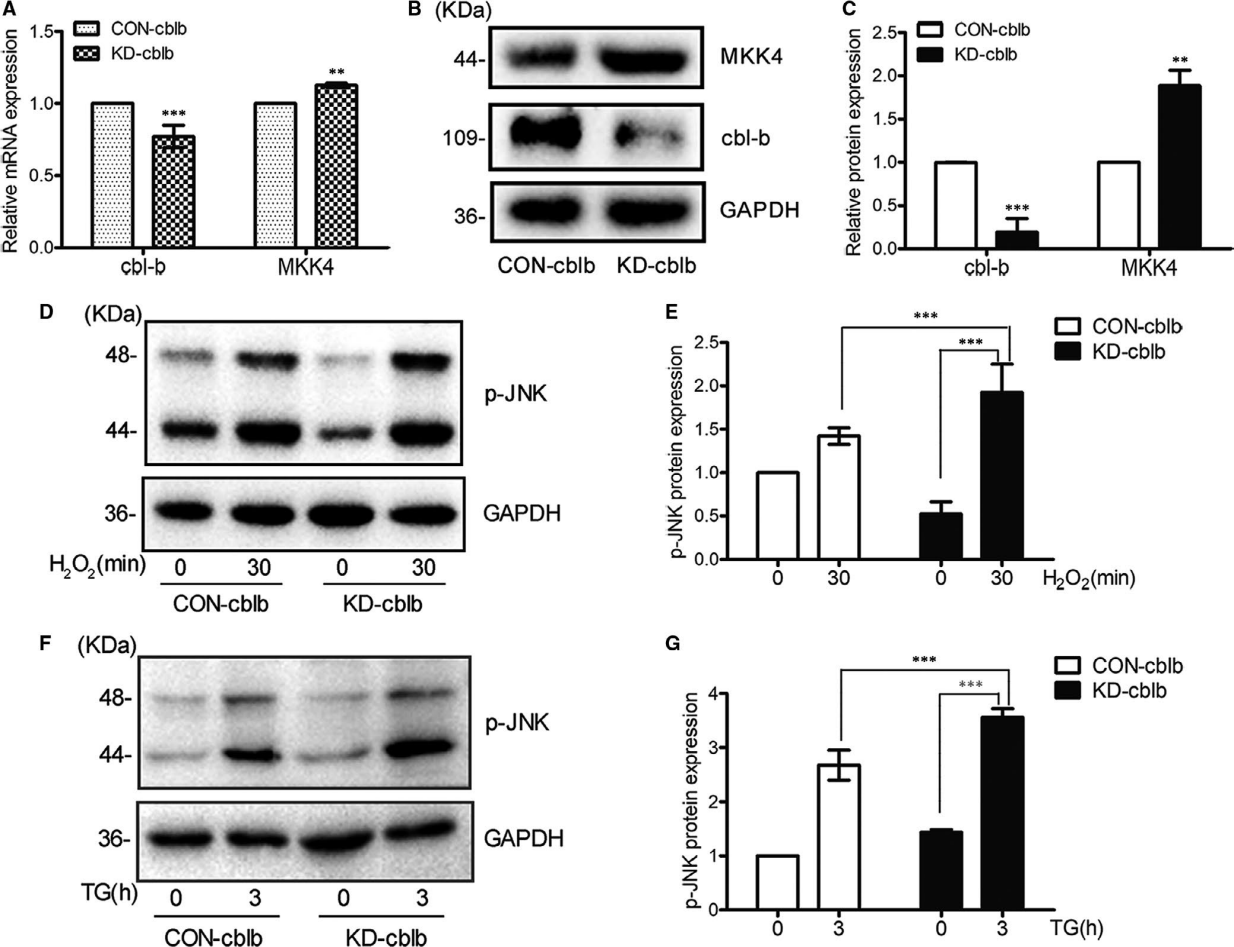
Cbl‐b expression negatively correlates with MKK4 protein level and p‐JNK level upon H_2_O_2_ or TG treatment in OV cells. A, OV cells were infected with Lentivirus encoding shRNA targeting cbl‐b or encoding scramble shRNA. Real‐time quantitative PCR was exploited to test the mRNA levels of cbl‐b and MKK4 in KD‐cbl‐b and CON‐cbl‐b. B and C, Western blotting was applied to detect the protein levels of cbl‐b and MKK4 in KD‐cbl‐b and CON‐cbl‐b. D and E, the protein levels of p‐JNK were exhibited in CON‐cbl‐b and KD‐cbl‐b cells in response to 100 μmol/L H_2_O_2_ at 0 or 30 min. F and G, the protein levels of p‐JNK were exhibited in CON‐cbl‐b and KD‐cbl‐b cells in response to 0.5 μmol/L TG at 0 or 3 h. The data represented the means of three independent experiments;**P < .01 and ***P < .001 vs ns; error bars indicate SD

To further confirm that cbl‐b modulates MKK4 protein expression in MIN6 cells, we applied RNA interference scheme to knock down cbl‐b expression in OV cells. Figure 5A‐C showed that knocking down cbl‐b expression resulted in increased MKK4 protein level. Knocking down cbl‐b expression resulted in about 20% and 90% increase in mRNA and protein levels of MKK4, respectively; therefore, the increased MKK4 protein level was not largely because of MKK4 transcription. Western blotting showed that TG or H_2_O_2_ treatment resulted in increased p‐JNK level in cbl‐b KD cells compared with that in control cells (Figure 5D‐G).

### 3.6 NR4A1 enhances the transactivation of *cbl‐b* promoter via physical association

It was reported that NR4A1 enhanced the *cbl‐b* promoter transactivation in human cells.^41^ We tested whether NR4A1 directly regulates *cbl‐b* transactivation in mouse cells regarding that humans and mice have some differences in promoter sequences.

**Figure 6:**
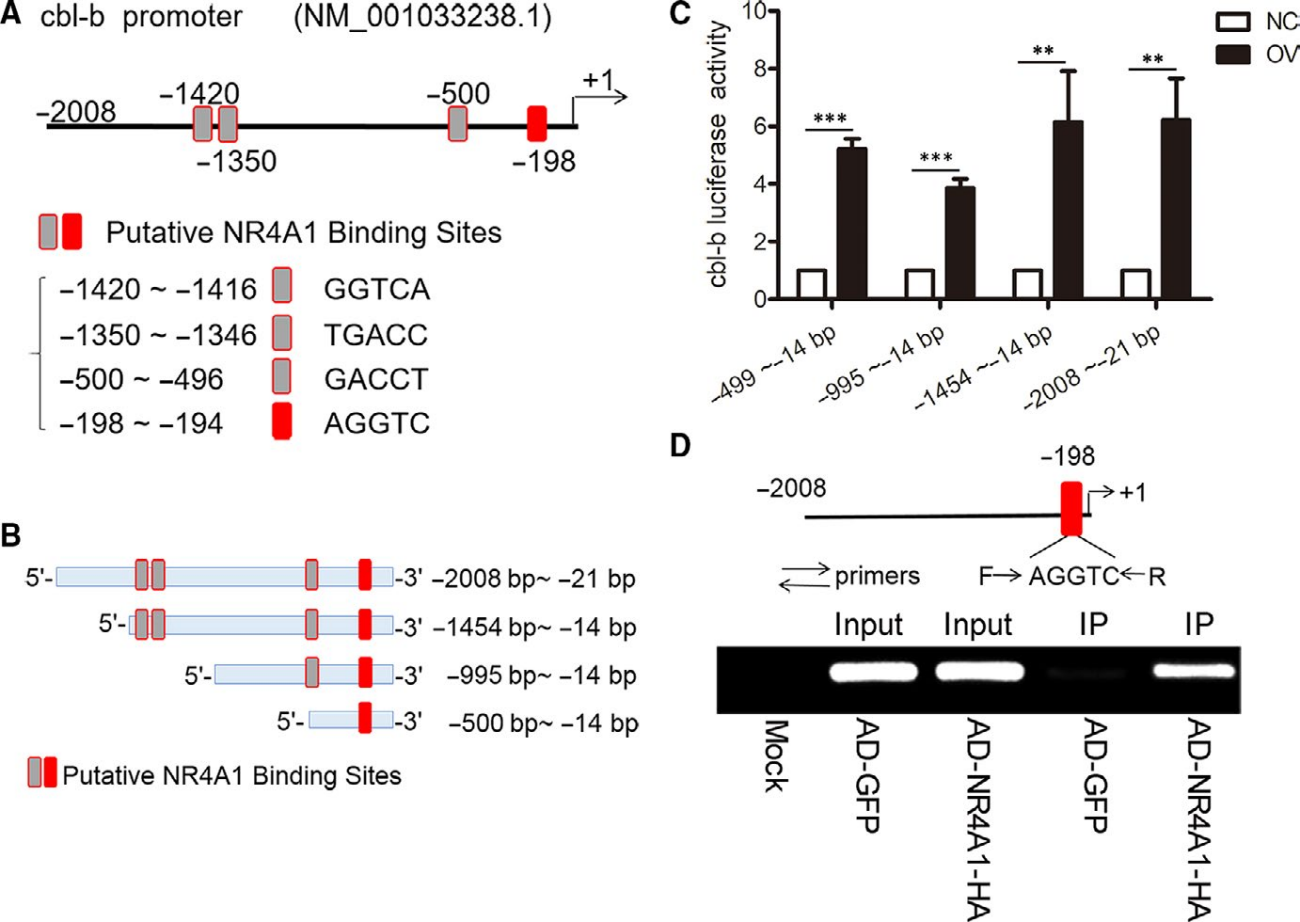
NR4A1 enhances the transactivation of cbl‐b promoter via physical association. A, a sketch applied to show the putative binding sites of NR4A1 in cbl‐b promoter. B, a diagram for cbl‐b promoters with different lengths was designed for luciferase reporter construction. C, the relative luciferase activity of cbl‐b promoters with different lengths was detected in both OV and NC cells. D, ChIP analysis. Chromatin DNA fragments combined with exogenous NR4A1‐HA were pulled down by a monoclonal antibody of HA; thereafter, a pair of primers was applied to amplify the fragment of the cbl‐b promoter containing a NR4A1 (or NBRE) binding site from the ChIP product. The data represented the means of three independent experiments; **P < .01 and ***P < .001 vs ns; error bars indicate SD

The promoter sequence of cbl‐b between −14 and −2008 has four putative NR4A1 binding sites as shown in Figure 6A. We cloned four *cbl‐b* luciferase reporters with different DNA lengths as shown in Figure 6B. We transfected the four cbl‐b luciferase reporter plasmids into OV and NC cells, the dual‐luciferase assay results showed that NR4A1 was able to enhance the luciferase activity of the four *cbl‐b* reporters with different lengths, and the shortest promoter sequence of *cbl‐b* (−14 to −499) was no less effective than the longer promoters (−14 to −995, −14 to −1454 and −22 to −2008) (Figure 6C). Namely, NR4A1 might modulate the promoter sequence at −14 to −499.

To verify whether NR4A1 associates with *cbl‐b* promoter, we infected MIN6 cells with adenovirus encoding NR4A1‐HA, and after the infection, the cells were applied for ChIP assay. The ChIP products obtained were applied for PCR examination. As shown in Figure 6D, the primers designed were covered the putative binding site at −118 to −251 bp within −14 to −499. The results in Figure 6D exhibited that after ChIP pulled down by anti‐HA antibodies, the putative binding site at −118 to −251 bp was amplified successfully. These results indicated that NR4A1 can physically associate with *cbl‐b* promoter.

### 3.7 Confirmation of the impact of NR4A1 on cbl‐b expression in NR4A1‐knockout mice

**Figure 7:**
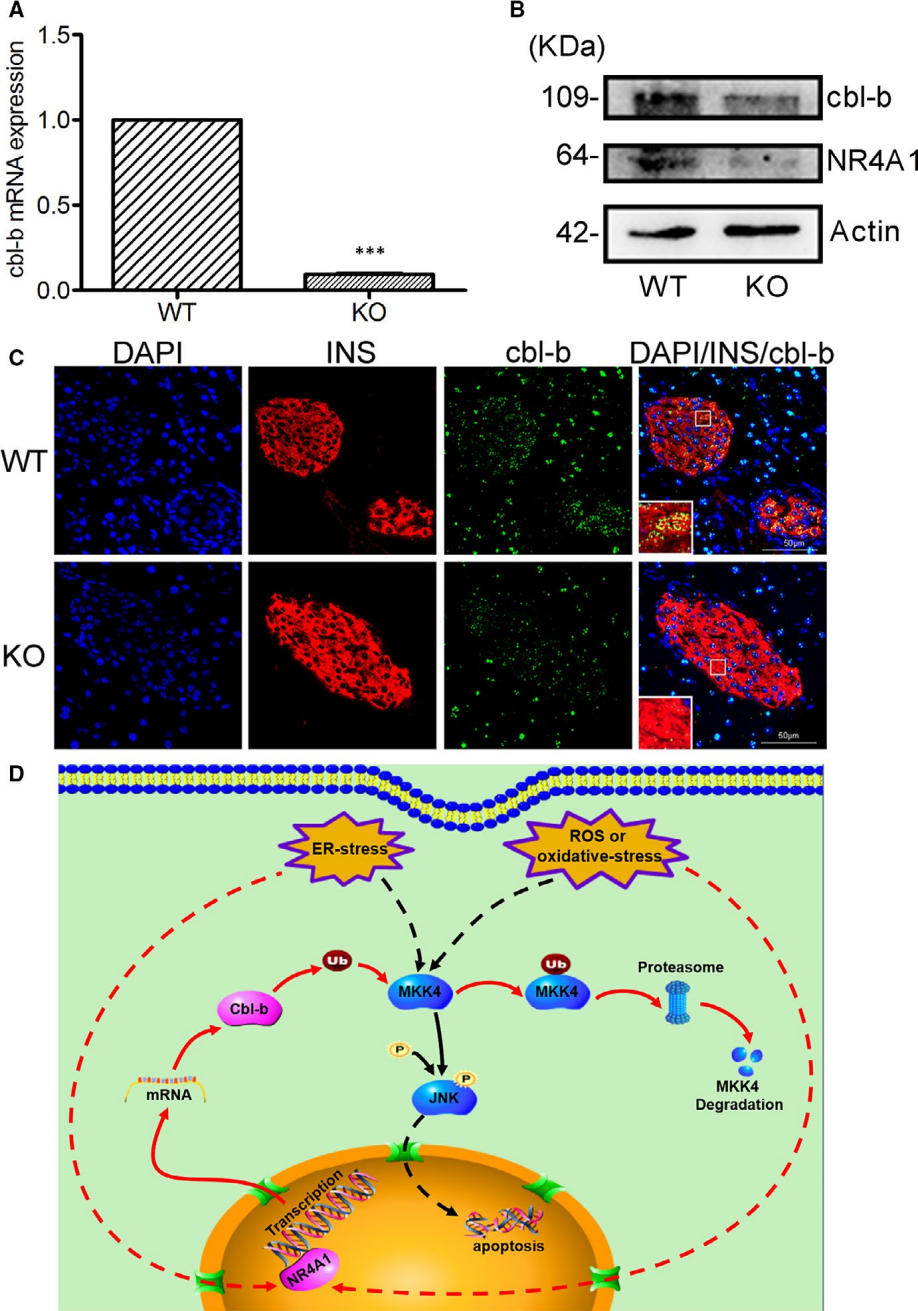
Confirmation of NR4A1 impacting cbl‐b expression in NR4A1‐KO mice. A, the relative mRNA levels of cbl‐b were determined by real‐time quantitative PCR in wild‐type mice and NR4A1 KO mice. B, the relative protein levels of cbl‐b and NR4A1 in pancreatic islets from WT or NR4A1 KO mice were determined by Western blotting. C, the colocalization of cbl‐b (green) with insulin (red) was dramatically decreased in the islets of pancreatic tissue from NR4A1‐KO mice compared with that from WT mice. The optical magnification of the image was 300×, and the larger square on the left bottom of each image was the enlarged image of the smaller section of the islet (the smaller square indicated within the islet). D, A graphic model for the mechanism that NR4A1 protects β‐cells from JNK phosphorylation induced by ER stress or ROS. The data represented the means of three independent experiments; ***P < .001 vs ns; error bars indicate SD

To confirm the conclusion that NR4A1 enhances the expression of cbl‐b in vivo, we tested the expression of cbl‐b in NR4A1‐KO mice. We purified the islets from WT mice or NR4A1‐KO mice, and further extracted the RNA and protein for real‐time PCR and Western blotting. The mRNA expression of cbl‐b in NR4A1‐KO mice was much lower than that in WT mice (Figure 7A), whereas the cbl‐b protein level in NR4A1‐KO mice was also lower than that in WT mice (Figure 7B).

The immuno‐staining data showed that the colocalization of cbl‐b with insulin was dramatically decreased in the islets of pancreatic tissue from NR4A1‐KO mice compared with that from WT mice (Figure 7C), which indicated that cbl‐b protein expression in pancreatic β‐cells was significantly reduced in NR4A1‐KO mice.

### 3.8 Summary of the results

Figure 7D is a graphic model for the mechanism that NR4A1 protects β‐cells from ER stress or ROS‐induced JNK phosphorylation. In β‐cells, ROS or ER stress results in JNK phosphorylation and consequently results in apoptosis. ROS or ER stress also induces NR4A1 expression, whereas NR4A1 enhances the expression of cbl‐b and further increases the degradation of MKK4, which results in reduced JNK phosphorylation. The fate of the pancreatic β‐cells is possibly determined by the balance between p‐JNK and cbl‐b.

In short, NR4A1 reduces p‐JNK level by indirectly down‐regulating the availability of its upstream MKK4, therefore, reduces the possibility of pancreatic β‐cell apoptosis.

## 4 DISCUSSION

Hyperglycaemia and hyperlipidaemia result in ER stress and/or increased ROS in pancreatic β‐cells, and cause β cells damage and apoptosis.^1^, ^2^, ^3^, ^4^, ^5^, ^6^ Pancreatic β‐cell loss plays an essential role in the pathogenesis of type 2 diabetes. Our pancreatic β‐cells are always challenged with hyperglycaemia and/or hyperlipidaemia after meal, but they are always able to survive over the subsequent cellular stress by an unknown “protective network” with some protective molecules. If the “protective network” is weak, consequently, the pancreatic β‐cells will be damaged or even undergo apoptosis. To search the molecules for pancreatic β‐cell protection under cellular stress is important for type 2 diabetes prevention.

It was reported that ER stress or ROS resulted in increased JNK activation or phosphorylation in various cells ^31^, ^32^, ^33^, ^35^ and activated JNK further triggered the signalling pathways to apoptosis.^36^, ^37^, ^38^, ^39^ We confirmed that MIN6 cells were treated with TG or H_2_O_2_ resulted in increased JNK phosphorylation. Some researcher applied JNK phosphorylation inhibitor which partially reversed the ER stress or ROS‐induced apoptosis in Bcr/Abl‐positive chronic myelogenous leukaemia cells,^45^ which indicated that JNK phosphorylation was critical for ER stress or ROS‐induced apoptosis.^46^

As for the mechanisms of ROS or ER stress induced JNK activation, many research articles reported that ROS induced ER stress, which has been popularly accepted.^47^, ^48^, ^49^ Previously, we tested the survival of pancreatic beta cell (MIN6 cells) in response to TG (ER stress inducer) or H_2_O_2_ (a ROS molecule) and by comparison we found H_2_O_2_ resulted in apoptosis in a shorter time duration compared with TG.^4^, ^5^ As the induction of JNK activation by H_2_O_2_ was much quicker than that by TG, it was reasonable that we speculated that ROS induced JNK activation via ER stress and another unknown parallel pathway.

NR4A1 (synonym as Nur77) was a multi‐factor response molecule and an anti‐stress factor.^7^, ^8^, ^9^ NR4A1 might have different roles in different tissues or different organs. People applied NR4A1 KO mice to test the pathophysiological changes in different research areas. For metabolism, Chao L C reported that NR4A1 KO mice fed with high‐fat diet were much easier to become obese compared with WT mice^50^; Pei L reported that NR4A1 had a role in gluconeogenesis^51^; Zhan Y Y reported that nur77 regulates LKB1 localization and thus modulates AMPK activation.^52^ For immunity system, some reporters showed that NR4A1 might have negative effect on T cell activation.^53^, ^54^

Previously, we found that the pancreatic cells from NR4A1 KO mice islets were more sensitive to TG or H_2_O_2_‐induced apoptosis compared with that from WT mice.^4^, ^5^ We observed that high‐fat diet feeding resulted in increased 8‐OHdG (a senescence marker) level and apoptosis rate in pancreatic cells from NR4A1 KO mice compared with that from WT mice.^5^ It is known that pancreatic cell senescence or/and pancreatic cell loss results in diabetes. We further figured out that NR4A1 directly or indirectly enhances the expression of some anti‐apoptotic proteins, such as survivin, WT1, BCL2, SOD1 and GPX1.^4^, ^5^, ^42^ These anti‐apoptotic proteins plus some other NR4A1‐up‐regulated molecules might form a protective network to work together to protect pancreatic β cells. Without the NR4A1‐based protective network, the pancreatic β cells would be in great risk or danger. We are still exploring other possible members of NR4A1‐up‐regulated protective network in β cells by exploiting pancreatic β cell line and NR4A1 KO mice.

We previously found that NR4A1 was inducible upon treatment with ER stress inducer (TG) or ROS (H_2_O_2_) in pancreatic β‐cells.^4^, ^5^, ^42^ Recently, we found that overexpression of NR4A1 in MIN6 cells resisted the JNK phosphorylation induced by TG or H_2_O_2_.

To study the mechanism of NR4A1 down‐regulating JNK phosphorylation, we analysed the key upstream kinases for JNK and we focused on two potential JNK kinases, MKK4 and MKK7 as reported. We further confirmed that MKK4 and MKK7 were two upstream kinases for JNK activation in MIN6 cells induced by H_2_O_2_ or TG. We compared the protein expression levels of the two molecules vs NR4A1 expression level and found that NR4A1 overexpression in MIN6 cells resulted in reduced expression of MKK4 rather than MKK7. As a transcription factor, usually NR4A1 enhances the expression of a gene. How could NR4A1 reduce the protein expression of MKK4? There should be two possible ways for the reduction in a specific protein expression besides the transcription activity altering: One is to interfere the protein translation, and the other is to modulate the stability of the protein. We could not rule out that NR4A1 is able to reduce MKK4 protein translation via some special paths. But we did predict that NR4A1 might have the effect on the stability of MKK4 protein via an indirect way. Most proteins' stability is closely related to their ubiquitination level and proteasome degradation, which lies in the protein level of substrate‐specific E3 ligase. We verified that the loss of MKK4 protein by NR4A1 overexpression was proteasome‐dependent by using a specific drug MG132.

Thus, we searched the literatures and found that the NR4A1 was able to enhance the expression of three E3 ligases.^19^, ^40^, ^41^ However, we only found cbl‐b mRNA level was increased in NR4A1 overexpression MIN6 cells. We further checked the protein expression of cbl‐b in NR4A1 overexpression cells (OV cells), as expected that overexpressing NR4A1 resulted in enhanced protein expression of cbl‐b. To confirm cbl‐b was able to target MKK4, we knocked down the cbl‐b expression in NR4A1 overexpression cells (OV cells) and found knocking‐down expression of cbl‐b in OV cells, resulted in increased MKK4 protein level and increased p‐JNK level upon treatment with TG or H_2_O_2_. It was clear that cbl‐b protein expression was reversely correlated with the MKK4 protein level or p‐JNK level. Our data indicated that cbl‐b was a down‐stream molecule of NR4A1 to down‐regulate MKK4 protein level in MIN6 cells.

As for cbl‐b, Keane et al firstly cloned this gene. Cbl‐b, with homology to the c‐cbl proto‐oncogene, has a proline‐rich domain, a nuclear localization signal, a C3HC4 zinc finger and a putative leucine zipper. Their data suggested that cbl‐b gene encodes a protein, which can interact with signal transduction proteins to regulate their function or to be regulated by them.^55^ As cbl‐b protein was reportedly found to contain a highly conserved RING finger domain with E3 ligase catalytic activity, it has been predicted that its biological functions are mediated via protein ubiquitination in diverse cellular signalling pathways. Such as, cbl‐b, as E3 ubiquitin ligase, negatively regulates CLR‐mediated antifungal innate immunity.^56^ Upon nerve growth factor (NGF) stimulation, recruitment of Cbl‐b promotes tropomyosin‐related kinase A (TrkA) ubiquitylation and degradation. Cbl‐b limits NGF‐TrkA signalling to control the length of neurites.^57^ In macrophages, Cbl‐b suppresses saturated FA‐induced Toll‐like receptor 4 (TLR4) signalling by ubiquitination and degradation of TLR4. Cbl‐b deficiency could exaggerate HFD‐induced insulin resistance through saturated FA‐mediated macrophage activation.^58^ We first discovered that cbl‐b was able to degrade MKK4 through the ubiquitination‐proteasome pathway, and thus to attenuate JNK phosphorylation in response to ER stress or ROS.

We further studied the possible mechanism(s) by which NR4A1 may up‐regulate cbl‐b expression. It was reported that NR4A1 enhanced *cbl‐b* promoter transactivation in human cells. To verify whether NR4A1 directly enhances *cbl‐b* transactivation in mice β‐cells, we exploited luciferase assay and ChIP assay. We further found NR4A1 was able to enhance the transactivation activity of *cbl‐b* promoter and NR4A1 was able to physically associate with *cbl‐b* at some putative targeting sequence. What we detected is the binding sites of NR4A1 in *cbl‐b* promoter regulatory element in mouse cells were not the same as people found in human cells as reported.

In addition to our in vitro study, we further found the mRNA level or protein level of cbl‐b was markedly reduced in the islets from NR4A1‐KO mice compared with that from WT mice, which was further confirmed that NR4A1 was a positive factor for cbl‐b expression in mice pancreatic islets. As shown in insulin immuno‐staining data, the majority of cells (about 85%‐90%) in pancreatic islets were β‐cells. Therefore, the mRNA and protein expression levels of cbl‐b detected from islets reflected the status of pancreatic β‐cells to a large extent. To further clarify NR4A1 impacting cbl‐b protein expression in β‐cells, we applied insulin and cbl‐b double immuno‐staining scheme to show the colocalization of the two molecules in β‐cells. Our immune‐staining data showed that the protein level of cbl‐b in pancreatic β‐cells of NR4A1‐KO mice was markedly reduced compared with that of WT mice. This evidence confirmed that NR4A1 impacted the expression of cbl‐b in mice pancreatic β‐cells, which was in concert with what we found in vitro.

In summary, our data demonstrated that NR4A1 could repress cellular ER stress or ROS‐induced JNK activation via enhancing cbl‐b expression to degrade MKK4 in pancreatic β‐cells. Namely, NR4A1 down‐regulates p‐JNK level through NR4A1/cbl‐b↑/MKK4↓/p‐JNK↓ pathway. Our study indicates NR4A1 might be a major player in the protective network of pancreatic β‐cells, and our finding might provide clues for β‐cell protection and diabetes prevention.

## CONFLICT OF INTEREST

No potential conflict of interest relevant to this article exists.

## AUTHOR CONTRIBUTIONS

**Xiangdong Wang:** Conceptualization (lead); Funding acquisition (lead); Investigation (lead); Resources (lead); Supervision (lead); Validation (lead); Visualization (equal); Writing‐original draft (lead); Writing‐review & editing (equal). **Zeqing Pu:** Data curation (equal); Formal analysis (equal); Methodology (equal); Software (equal); Visualization (equal); Writing‐original draft (equal). **Dong Liu:** Data curation (equal); Formal analysis (equal); Methodology (equal); Software (equal). **Hanse Pablick Patherny Lobo Mouguegue:** Data curation (equal); Investigation (equal); Methodology (equal). **Chengwen Jin:** Data curation (equal); Investigation (equal); Methodology (equal); Software (equal). **Esha Sadiq:** Data curation (equal); Methodology (equal). **Dandan Qin:** Data curation (equal); Formal analysis (equal); Methodology (equal); Software (equal). **Tianfu Yu:** Data curation (equal); Formal analysis (equal); Methodology (equal); Software (equal). **Chen Zong:** Data curation (equal); Methodology (equal); Software (equal). **Jicui Chen:** Methodology (equal); Software (equal); Visualization (equal). **Ruxing Zhao:** Data curation (equal); Formal analysis (equal); Funding acquisition (supporting); Resources (equal). **Jingyu Lin:** Methodology (equal). **Jie Cheng:** Methodology (equal). **xiao yu:** Investigation (supporting); Methodology (equal). **Xia Li:** Conceptualization (equal); Funding acquisition (equal); Investigation (equal); Validation (equal). **Yuchao Zhang:** Methodology (equal); Software (equal). **Yuantao Liu:** Conceptualization (equal); Writing‐original draft (equal). **Bo Qing Guan:** Conceptualization (supporting); Investigation (equal).

## Data Availability

All data generated or analysed during this study are included in this published article, and the original data are always available to the editor and readers upon request.

## References

[1] Butler AE , Janson J , Bonner‐Weir S , Ritzel R , Rizza RA , Butler PC . β‐cell deficit and increased β‐cell apoptosis in humans with type 2 diabetes. Diabetes. 2003;52:102‐110. 10.2337/diabetes.52.1.102 12502499

[2] Biarnés M , Montolio M , Nacher V , Raurell M , Soler J , Montanya E . β‐cell death and mass in syngeneically transplanted islets exposed to short‐ and long‐term hyperglycemia. Diabetes. 2002;51:66‐72. 10.2337/diabetes.51.1.66 11756324

[3] Maris M , Robert S , Waelkens E , et al. Role of the saturated nonesterified fatty acid palmitate in beta cell dysfunction. J Proteome Res. 2013;12:347‐362. 10.1021/pr300596g 23170928

[4] Yu C , Cui S , Zong C , et al. The orphan nuclear receptor NR4A1 protects pancreatic β‐cells from endoplasmic reticulum (ER) stress‐mediated apoptosis. J Biol Chem. 2015;290:20687‐20699. 10.1074/jbc.M115.654863 26157144 PMC4543630

[5] Zong C , Qin D , Yu C , et al. The stress‐response molecule NR4A1 resists ROS‐induced pancreatic β‐cells apoptosis via WT1. Cell Signal. 2017;35:129‐139. 10.1016/j.cellsig.2017.03.012 28342843

[6] Prause M , Christensen DP , Billestrup N , Mandrup‐Poulsen T . JNK1 protects against glucolipotoxicity‐mediated beta‐cell apoptosis. PLoS One. 2014;9:e87067 10.1371/journal.pone.0087067 24475223 PMC3901710

[7] Berriel Diaz M , Lemke U , Herzig S . Discovering orphans' sweet secret: NR4A receptors and hepatic glucose production. Cell Metab. 2006;4:339‐340. 10.1016/j.cmet.2006.10.005 17084708

[8] Li X , Wang Z , Zheng Y , et al. Nuclear receptor Nur77 facilitates melanoma cell survival under metabolic stress by protecting fatty acid oxidation. Mol Cell. 2018;69(3):480‐492.e7. 10.1016/j.molcel.2018.01.001 29395065

[9] Hu M , Luo Q , Alitongbieke G , et al. Celastrol‐induced Nur77 interaction with TRAF2 alleviates inflammation by promoting mitochondrial ubiquitination and autophagy. Mol Cell. 2017;66(1):141‐153.e6. 10.1016/j.molcel.2017.03.008 28388439 PMC5761061

[10] Walker NPC , Perlmann T , Wang Z , et al. Structure and function of Nurr1 identifies a class of ligand‐independent nuclear receptors. Nature. 2003;423:555‐560. 10.1038/nature01645 12774125

[11] Mohan HM , Aherne CM , Rogers AC , Baird AW , Winter DC , Murphy EP . Molecular pathways: the role of NR4A orphan nuclear receptors in cancer. Clin Cancer Res. 2012;18:3223‐3228. 10.1158/1078-0432.CCR-11-2953 22566377

[12] Kurland IJ , Pei L , Waki H , Tontonoz P , Vaitheesvaran B , Wilpitz DC . NR4A orphan nuclear receptors are transcriptional regulators of hepatic glucose metabolism. Nat Med. 2006;12:1048‐1055. 10.1038/nm1471 16906154

[13] Seo H , Chen J , González‐Avalos E , et al. TOX and TOX2 transcription factors cooperate with NR4A transcription factors to impose CD8+ T cell exhaustion. Proc Natl Acad Sci USA. 2019;116:12410‐12415. 10.1073/pnas.1905675116 31152140 10.1073/pnas.1905675116 PMC6589758

[14] Boddupalli CS , Nair S , Gray SM , et al. ABC transporters and NR4A1 identify a quiescent subset of tissue‐resident memory T cells. J Clin Investig. 2016;126:3905‐3916. 10.1172/JCI85329 27617863 PMC5096804

[15] Hanna RN , Carlin LM , Hubbeling HG , et al. The transcription factor NR4A1 (Nur77) controls bone marrow differentiation and the survival of Ly6C‐ monocytes. Nat Immunol. 2011;12:778‐785. 10.1038/ni.2063 21725321 PMC3324395

[16] Cheng Z , Völkers M , Din S , et al. Mitochondrial translocation of Nur77 mediates cardiomyocyte apoptosis. Eur Heart J. 2011;32:2179‐2188. 10.1093/eurheartj/ehq496 21228009 PMC3164102

[17] Freire P , Conneely O . NR4A1 and NR4A3 restrict HSC proliferation via reciprocal regulation of C/EBP alpha and inflammatory signaling. Blood. 2018;131:1081‐1093. 10.1182/blood-2017-07-795757 29343483 PMC5863701

[18] Deutsch AJA , Rinner B , Wenzl K , et al. NR4A1‐mediated apoptosis suppresses lymphomagenesis and is associated with a favorable cancer‐specific survival in patients with aggressive B‐cell lymphomas. Blood. 2014;123:2367‐2377. 10.1182/blood-2013-08-518878 24553175

[19] Mullican S , Zhang S , Konopleva M , et al. Abrogation of nuclear receptors Nr4a3 and Nr4a1 leads to development of acute myeloid leukemia. Nat Med. 2007;13:730‐735. 10.1038/nm1579 17515897

[20] Ke N , Claassen G , Yu D , et al. Nuclear hormone receptor NR4A2 is involved in cell transformation and apoptosis. Cancer Res. 2004;64:8208‐8212. 10.1158/0008-5472.CAN-04-2134 15548686

[21] Suzuki S , Suzuki N , Mirtsos C , et al. Nur77 as a survival factor in tumor necrosis factor signaling. Proc Natl Acad Sci USA. 2003;100(14):8276‐8280. 10.1073/pnas.0932598100 12815108 PMC166219

[22] Lee S , Lee S , Jin U , et al. The orphan nuclear receptor NR4A1 (Nur77) regulates oxidative and endoplasmic reticulum stress in pancreatic cancer cells. Mol Cancer Res. 2014;12:527‐538. 10.1158/1541-7786.mcr-13-0567 24515801 PMC4407472

[23] Lee S , Li X , Hedrick E , et al. Diindolylmethane analogs bind NR4A1 and Are NR4A1 antagonists in colon cancer cells. Mol Endocrinol. 2014;28:1729‐1739. 10.1210/me.2014-1102 25099012 PMC4179635

[24] Lee S , Abdelrahim M , Yoon K , et al. Inactivation of the orphan nuclear receptor TR3/Nur77 inhibits pancreatic cancer cell and tumor growth. Cancer Res. 2010;70:6824‐6836. 10.1158/0008-5472.CAN-10-1992 20660371 PMC2988472

[25] Oh S , Oh J , Lee C , et al. Expression of Twist2 is controlled by T‐cell receptor signaling and determines the survival and death of thymocytes. Cell Death Differ. 2016;23:1804‐1814. 10.1038/cdd.2016.68 27391798 PMC5071571

[26] Yang H , Zhan Q , Wan YY . Enrichment of Nur77 mediated by retinoic acid receptor β leads to apoptosis of human hepatocellular carcinoma cells induced by fenretinide and histone deacetylase inhibitors. Hepatology. 2011;53:865‐874. 10.1002/hep.24101 21319187 PMC3077573

[27] Chen J , Fiskus W , Eaton K , et al. Cotreatment with BCL‐2 antagonist sensitizes cutaneous T‐cell lymphoma to lethal action of HDAC7‐Nur77‐based mechanism. Blood. 2009;113:4038‐4048. 10.1182/blood-2008-08-176024 19074726

[28] Shen H , Liu Z . JNK signaling pathway is a key modulator in cell death mediated by reactive oxygen and nitrogen species. Free Rad Biol Med. 2006;40:928‐939. 10.1016/j.freeradbiomed.2005.10.056 16540388

[29] Win S , Than TA , Min RWM , Aghajan M , Kaplowitz N . c‐Jun N‐terminal kinase mediates mouse liver injury through a novel Sab (SH3BP5)‐dependent pathway leading to inactivation of intramitochondrial Src. Hepatology. 2016;63:1987‐2003. 10.1002/hep.28486 26845758 PMC4874901

[30] Seki E , Brenner DA , Karin M . A Liver full of JNK: signaling in regulation of cell function and disease pathogenesis, and clinical approaches. Gastroenterology. 2012;143:307‐320. 10.1053/j.gastro.2012.06.004 22705006 PMC3523093

[31] Ibrahim SH , Gores GJ . Who pulls the trigger: JNK activation in liver lipotoxicity? J Hepatol. 2011;2012(56):17‐19. 10.1016/j.jhep.2011.04.017 PMC3297468 21703172

[32] Czaja MJ . JNK regulation of hepatic manifestations of the metabolic syndrome. Trends Endocrinol Metab. 2010;21:707‐713. 10.1016/j.tem.2010.08.010 20888782 PMC2991513

[33] Holzer R , Park E , Li N , et al. Saturated fatty acids induce c‐Src clustering within membrane subdomains, leading to JNK activation. Cell. 2011;147:173‐184. 10.1016/j.cell.2011.08.034 21962514 PMC3295636

[34] Liu J , Lin A . Role of JNK activation in apoptosis: a double‐edged sword. Cell Res. 2005;15(1):36‐42. 10.1038/sj.cr.7290262 15686625

[35] Win S , Than TA , Le BHA , García‐Ruiz C , Fernandez‐Checa JC , Kaplowitz N . Sab (Sh3bp5) dependence of JNK mediated inhibition of mitochondrial respiration in palmitic acid induced hepatocyte lipotoxicity. J Hepatol. 2015;62:1367‐1374. 10.1016/j.jhep.2015.01.032 25666017 PMC4439305

[36] Taylor CA , Zheng Q , Liu Z , Thompson JE . Role of p38 and JNK MAPK signaling pathways and tumor suppressor p53 on induction of apoptosis in response to Ad‐eIF5A1 in A549 lung cancer cells. Mol Cancer. 2013;12:35 10.1186/1476-4598-12-35 23638878 PMC3660295

[37] Konishi H , Fujiya M , Tanaka H , et al. Probiotic‐derived ferrichrome inhibits colon cancer progression via JNK‐mediated apoptosis. Nat Commun. 2016;7:12365 10.1038/ncomms12365 27507542 PMC4987524

[38] Lin S , Hoffmann K , Gao C , Petrulionis M , Herr I , Schemmer P . Melatonin promotes sorafenib‐induced apoptosis through synergistic activation of JNK/c‐jun pathway in human hepatocellular carcinoma. J Pineal Res. 2017;62:e12398 10.1111/jpi.12398 28178378

[39] Song IS , Jun SY , Na H , et al. Inhibition of MKK7–JNK by the TOR signaling pathway regulator‐like protein contributes to resistance of HCC cells to TRAIL‐induced apoptosis. Gastroenterology. 2012;143:1341‐1351. 10.1053/j.gastro.2012.07.103 22841785

[40] Yang L , Zhao L , Gan Z , et al. Deficiency in RNA editing enzyme ADAR2 impairs regulated exocytosis. FASEB J. 2010;24:3720‐3732. 10.1096/fj.09-152363 20501795

[41] Li X , Wei W , Huynh H , Zuo H , Wang X , Wan Y . Nur77 prevents excessive osteoclastogenesis by inducing ubiquitin ligase Cbl‐b to mediate NFATc1 self‐limitation. eLife. 2015;4:e07217 10.7554/eLife.07217 26173181 PMC4518709

[42] Yang Y , Xie F , Qin D , et al. The orphan nuclear receptor NR4A1 attenuates oxidative stress‐induced β cells apoptosis via up‐regulation of glutathione peroxidase 1. Life Sci. 2018;203:225‐232. 10.1016/j.lfs.2018.04.027 29678743

[43] Gao W , Fu Y , Yu C , et al. Elevation of NR4A3 expression and its possible role in modulating insulin expression in the pancreatic beta cell. PLoS One. 2014;9:e91462 10.1371/journal.pone.0091462 24638142 PMC3956668

[44] Lin H , Lin Q , Liu M , et al. PKA/Smurf1 signaling‐mediated stabilization of Nur77 is required for anticancer drug cisplatin‐induced apoptosis. Oncogene. 2014;33:1629‐1639. 10.1038/onc.2013.116 23584473

[45] Mao X , Rong YUC , Hua Li W , Xin LW . Induction of apoptosis by shikonin through a ROS/JNK‐mediated process in Bcr/Abl‐positive chronic myelogenous leukemia (CML) cells. Cell Res. 2008;18:879‐888. 10.1038/cr.2008.86 18663379

[46] Timmins JM , Ozcan L , Seimon TA , et al. Calcium/calmodulin‐dependent protein kinase II links ER stress with Fas and mitochondrial apoptosis pathways. J Clin Investig. 2009;119:2925‐2941. 10.1172/jci38857 19741297 PMC2752072

[47] Park S , Lim W , Bazer FW , Song G . Apigenin induces ROS‐dependent apoptosis and ER stress in human endometriosis cells. J Cell Physiol. 2018;233(4):3055‐3065. 10.1002/jcp.26054 28617956

[48] Wang Y , Lee K , Lim M , Choi J . TRPV1 antagonist DWP05195 induces ER stress‐dependent apoptosis through the ROS‐p38‐CHOP pathway in human ovarian cancer cells. Cancers. 2020;12(6):1702 10.3390/cancers12061702 PMC7352786 32604833

[49] Lin M , Li L , Zhang Y , et al. Baicalin ameliorates H2O2 induced cytotoxicity in HK‐2 cells through the inhibition of ER stress and the activation of Nrf2 Signaling. Int J Mol Sci. 2014;15:12507‐12522. 10.3390/ijms150712507 25029541 PMC4139857

[50] Chao LC , Wroblewski K , Zhang Z , et al. Insulin resistance and altered systemic glucose metabolism in mice lacking Nur77. Diabetes. 2009;58:2788‐2796. 10.2337/db09-0763 19741162 PMC2780886

[51] Pei L , Waki H , Tontonoz P , Vaitheesvaran B , Wilpitz DC . NR4A orphan nuclear receptors are transcriptional regulators of hepatic glucose metabolism. Nat Med. 2006;12:1048‐1055. 10.1038/nm1471 16906154

[52] Zhan Y , Chen Y , Zhang Q , et al. The orphan nuclear receptor Nur77 regulates LKB1 localization and activates AMPK. Nat Chem Biol. 2012;8:897‐904. 10.1038/nchembio.1069 22983157

[53] Liu X , Wang Y , Lu H , et al. Genome‐wide analysis identifies NR4A1 as a key mediator of T cell dysfunction. Nature. 2019;567:525‐529. 10.1038/s41586-019-0979-8 30814730 PMC6507425

[54] Chen J , López‐Moyado IF , Seo H , et al. NR4A transcription factors limit CAR T cell function in solid tumours. Nature. 2019;567:530‐534. 10.1038/s41586-019-0985-x 30814732 PMC6546093

[55] Keane MM , Rivero‐Lezcano OM , Mitchell JA , Robbins KC , Lipkowitz S . Cloning and characterization of cbl‐b: A SH3 binding protein with homology to the c‐cbl proto‐oncogene. Oncogene. 1995;10:2367‐2377. 10.1016/0360-3016(95)93133-R 7784085

[56] Zhu L , Luo T , Xu X , et al. E3 ubiquitin ligase Cbl‐b negatively regulates C‐type lectin receptor‐mediated antifungal innate immunity. J Exp Med. 2016;213:1555‐1570. 10.1084/jem.20151932 27432944 PMC4986534

[57] Emdal KB , Pedersen A , Bekker‐Jensen DB , et al. Temporal proteomics of NGF‐TrkA signaling identifies an inhibitory role for the E3 ligase Cbl‐b in neuroblastoma cell differentiation. Sci Signal. 2015;8:ra40 10.1126/scisignal.2005769 25921289

[58] Abe T , Hirasaka K , Kagawa S , et al. Cbl‐b is a critical regulator of macrophage activation associated with obesity‐induced insulin resistance in mice. Diabetes. 2013;62:1957‐1969. 10.2337/db12-0677 23349502 PMC3661636

